# The Spectrum of Motor Disorders in Patients with Chronic Kidney Disease: Pathogenic Mechanisms, Clinical Manifestations, and Therapeutic Strategies

**DOI:** 10.3390/jcm15020537

**Published:** 2026-01-09

**Authors:** Patryk Jerzak, Jakub Mizera, Tomasz Gołębiowski, Magdalena Kuriata-Kordek, Mirosław Banasik

**Affiliations:** Clinical Department of Nephrology, Transplantation Medicine and Internal Diseases, Institute of Internal Diseases, Wroclaw Medical University, 50-556 Wroclaw, Poland; jakub.mizera.md@gmail.com (J.M.); tomasz.golebiowski@umw.edu.pl (T.G.); magdalena.kuriata-kordek@umw.edu.pl (M.K.-K.); miroslaw.banasik@umw.edu.pl (M.B.)

**Keywords:** motor disorders, chronic kidney disease, quality of life, dialysis, neuromuscular disease, restless legs syndrome

## Abstract

Motor disorders are increasingly recognized as a significant complication of chronic kidney disease (CKD), yet they remain underdiagnosed, undertreated, and often overlooked in clinical practice. Patients with CKD experience a broad spectrum of motor disturbances, including restless legs syndrome, myoclonus, flapping tremor, periodic limb movements in sleep, Parkinsonism, and peripheral neuropathy. These disorders arise from complex and often overlapping mechanisms such as uremic neurotoxicity, vascular injury, electrolyte and hormonal imbalances, or inflammatory processes, reflecting the systemic impact of impaired renal function on the central and peripheral nervous systems. The presence of motor disorders in CKD is associated with substantial clinical consequences for quality of life, contributing to impaired mobility, persistent insomnia, daytime fatigue, higher fall risk, and diminished independence. Moreover, these disturbances have been linked to increased cardiovascular morbidity and mortality, further exacerbating the already high burden of disease in this population. Current management approaches focus on optimizing kidney function through dialysis or transplantation, pharmacological therapies such as dopaminergic agents, gabapentinoids, and iron supplementation, as well as non-pharmacological interventions including structured exercise programs and sleep hygiene measures. Despite these strategies, robust evidence on long-term outcomes, comparative effectiveness, and optimal treatment algorithms remains limited. Greater recognition of the clinical impact of motor disorders in CKD, combined with targeted research efforts, is urgently needed to improve patient-centered outcomes and guide evidence-based care.

## 1. Introduction

Motor disorders (MDs) are increasingly recognized as a significant but often overlooked complication of chronic kidney disease (CKD). They range from mild coordination and fine motor deficits to more disabling conditions such as tremor, bradykinesia, and gait abnormalities [1].

This matter is particularly important considering that chronic kidney disease, from its mild stages to end-stage renal disease, affects approximately 10% of the global population, with many individuals unaware of their progressively declining kidney function [2].

Epidemiological data reveal a high prevalence of movement disorders in the CKD population. Restless legs syndrome (RLS), one of the most studied conditions, affects 20–40% of patients, which is 2–3 times more frequent than in the general population [3]. Other notable motor complications include periodic limb movements in sleep (17%), flapping tremor (20.9%), and myoclonus (24.1%), while Parkinsonism occurs in approximately 2.8% of cases when it comes to the most advanced CKD cases requiring dialysis [4]. Furthermore, in end-stage renal disease, patients undergoing dialysis are even more susceptible to motor disorders, which are frequently accompanied by weakness, reduced endurance, and progressive physical decline [5].

The mechanisms driving MDs in patients with CKD are complex and multifactorial. Key contributors include the accumulation of uremic toxins, which lead to disruption of the blood–brain barrier and endothelial damage. All these factors can contribute to dysfunction of subcortical structures such as the basal ganglia, particularly important in proper motor function [1]. These are often accompanied by systemic features of CKD such as vascular stiffness, chronic inflammation, iron deficiency, and other metabolic disturbances, which further worsen overall patient condition [6].

The impact of MDs in CKD extends well beyond motor impairment. They are associated with higher mortality, increased cardiovascular risk, and diminished quality of life [7]. For instance, impaired coordination heightens the risk of falls and adverse cardiovascular outcomes, while RLS has been linked to insomnia, depression, and excess mortality [3]. These complications tend to be more prevalent and severe in advanced CKD and among dialysis patients, where muscle wasting and fatigue are particularly common [8].

Management of MDs in CKD is evolving. Pharmacological treatments such as dopaminergic agents and gabapentinoids have demonstrated benefits, while non-drug strategies, particularly exercise programs during dialysis, are gaining support for improving functional outcomes and quality of life [3,9]. Still, questions remain regarding optimal intervention timing, dosing, and individualization of care [10].

Given their clinical significance, motor disorders in CKD require early recognition and a comprehensive treatment approach due to the strong association with adverse outcomes, worse quality of life, and even increased mortality [11]. While our understanding of these conditions has been growing, significant gaps remain in both prevention and treatment.

This review aims to provide an integrated overview of motor disorders in CKD, covering their prevalence, pathophysiological basis, clinical consequences, and current management strategies. By synthesizing recent findings, we seek to highlight both established insights and emerging therapeutic directions, with the goal of improving outcomes for patients facing the dual burden of CKD and motor dysfunction.

## 2. Materials and Methods

The authors conducted an extensive literature review, covering articles published up until September 2025. A variety of keyword combinations, including “motor disorders”, “chronic kidney disease”, “end-stage renal disease”, “dialysis”, “patient survival” and “quality of life” were used to guide the search. Medical databases such as PubMed, Google Scholar, Scopus, Web of Science, and Embase were utilized to ensure comprehensive coverage of relevant literature.

To guide the review and literature search, the following questions were formulated regarding the incidence, diagnosis and management of motor disorders in patients with chronic kidney disease:

- RQ1: What are the most prevalent motor disorders in CKD patients across different disease stages and treatment modalities, and how do these rates compare to the general population?
- RQ2: How do motor disorders in CKD impact quality of life, risk of falls, cardiovascular outcomes, and mortality?
- RQ3: What is the comparative effectiveness of pharmacological versus non-pharmacological interventions in managing motor disorders in CKD patients?
- RQ4: Can early detection and targeted rehabilitation programs prevent the progression of motor dysfunction in CKD?

Initially, the articles were screened based on their abstracts, with only those deemed most relevant subjected to a more detailed evaluation. Abstracts in languages other than English were excluded from the review. The literature search was conducted by a team of authors, who screened approximately 10,000 records. Eventually, 70 relevant studies specifically addressing motor disorders in patients with chronic kidney disease were included in the final review.

## 3. Pathophysiological Mechanisms Underlying MDs in CKD

The development of motor disorders in chronic kidney disease is driven by multiple, often overlapping, pathophysiological processes. Most of all, CKD can cause damage and disrupt the function of neural cells through two major mechanisms: metabolic and vascular [12]. Both mechanisms can lead to damage in the cortical and subcortical regions of the brain [13]. Notably, subcortical injury is particularly significant in the development of MDs, as these conditions frequently involve pathology within the basal ganglia and other subcortical brain nuclei [14,15].

### 3.1. Retention of Uremic Toxins—Metabolic Pathway

Multiple studies have already demonstrated the link between accumulation of basic uremic toxins, such as urea, during renal insufficiency and disturbances in neuronal osmotic regulation and metabolic homeostasis within the basal ganglia [1]. More neurotoxic protein-bound solutes, including indoxyl sulphate (IS) and p-cresol sulphate (PCS), cross the blood–brain barrier via organic anion transporters and induce pronounced oxidative stress by impairing mitochondrial respiratory chain activity, resulting in elevated reactive oxygen species (ROS) production and subsequent astrocytic apoptosis [16]. These toxins trigger neuroinflammation by activating the NLRP3 inflammasome in microglia and astrocytes, which promotes the maturation and release of proinflammatory cytokines such as interleukin-1β (IL-1β). Guanidino compounds disrupt basal ganglia function by blocking inhibitory neurotransmitter receptors (GABA_A and glycine receptors), leading to excitotoxic signalling and calcium overload within neurons. In addition, p-cresyl sulphate (PCS) damages the cerebrovascular endothelium by increasing the expression of matrix metalloproteinase-9 (MMP-9), an enzyme that breaks down tight junction proteins, thereby weakening the blood–brain barrier (BBB) and increasing its permeability [17,18]. Collectively, these mechanisms, including oxidative damage, mitochondrial dysfunction, neuroinflammation, and neurotransmitter imbalance, converge to disrupt basal ganglia neuronal viability and function.

### 3.2. Impaired Cerebral Perfusion—Vascular Pathway

In CKD, arterial stiffening results from a complex interplay of uraemia-induced metabolic disturbances and inflammation. Uremic toxins (e.g., phosphate, indoxyl sulphate, advanced glycation end-products) accumulate and, together with chronic oxidative stress and elevated cytokines, damage endothelial cells and promote vascular smooth muscle proliferation and fibrosis [19,20]. These processes drive elastin fragmentation, collagen deposition, and medial calcification in large arteries, markedly reducing their elasticity. The stiffened arteries lose their Windkessel function—systolic pressure rises and diastolic runoff falls—so cerebral perfusion becomes more pulsatile and pressure-dependent. In the brain’s microvasculature, this hemodynamic stress causes blood–brain barrier disruption and microvascular injury. Microglia are activated, and neurons endure chronic hypoxia and exposure to inflammatory mediators [21]. Over time, such neurovascular injury (neuronal apoptosis, synaptic dysfunction, and glial activation) can ultimately undermine motor pathways.

### 3.3. Other Pathomechanisms

#### 3.3.1. Electrolyte and Hormonal Disturbances

Chronic kidney disease can lead to a deranged electrolyte balance that directly injures neural cells and muscles. Hyperkalemia depolarizes axonal membranes, thereby increasing nerve excitability and contributing to the development of muscle dysfunction; however, it does not directly induce structural neuropathic changes [22]. Disturbed mineral metabolism (e.g., secondary hyperparathyroidism from calcium–phosphate imbalance) harms neuromuscular function as well; for example, dialysis patients sometimes show improved neural conduction after parathyroidectomy [22,23,24]. These observations, along with the finding that dietary potassium restriction can improve CKD-related neuropathy, suggest that uremic toxins, secondary hyperparathyroidism, and other electrolyte/mineral disturbances may play a significant role in the onset and progression of MDs in CKD.

#### 3.3.2. Sympathetic Overactivity and Abnormal Neurocirculatory Control

CKD also disrupts the neural regulation of blood flow during exercise and activity. Studies show that patients with CKD/ESRD have an exaggerated blood pressure response to exercise, even under mild exertion [25]. This hyper-pressor response reflects dysregulated sympathetic reflexes: although resting muscle sympathetic nerve activity (MSNA) is already elevated in CKD, CKD patients exhibit an abnormally high increase in MSNA during both isometric and rhythmic exercise when baroreflex constraints are removed [26]. Mechanoreceptor (mechanoreflex) input becomes overly sensitive, whereas metaboreflex-mediated sympathetic drives are blunted, and the normal process of functional sympatholysis (locally mediated vasodilation in working muscle) is impaired [25]. This neurocirculatory dysfunction limits muscle blood flow and oxygen delivery, contributing to exercise intolerance and motor impairment in CKD.

#### 3.3.3. aHUS (Atypical Hemolytic Uremic Syndrome) and Neurological Manifestations

aHUS is a thrombotic microangiopathy caused by uncontrolled activation of the alternative complement pathway and characterized by the triad of microangiopathic hemolytic anemia, thrombocytopenia, and acute kidney injury. The kidneys are the primary target; however, systemic involvement is common, particularly affecting the central nervous system. Symptoms are: headache, seizures, visual disturbances, focal neurological deficits, and coma in severe cases [27]. The introduction of anti-C5 agents such as eculizumab into clinical practice has enabled rapid and effective treatment of these neurological manifestations [28]. Early initiation of complement inhibition has been shown to improve both renal and neurological outcomes, reducing morbidity and mortality. Moreover, prompt therapy may prevent irreversible microvascular damage and long-term neurological sequelae. These findings underscore the importance of early diagnosis and timely therapeutic intervention in patients with suspected aHUS.

## 4. Prevalence and Types of Motor Disorders in CKD

Motor disorders in patients with CKD are characterized by a wide spectrum of clinical manifestations that can significantly affect daily functioning and overall health status. Among the most prevalent conditions are restless leg syndrome (RLS), myoclonus, flapping tremor, periodic limb movements in sleep (PLMS), Parkinsonism, and peripheral neuropathy, each of which contributes to reduced mobility, decreased quality of life, and a greater risk of disability [4]. Importantly, some of these disorders tend to improve when kidney function stabilizes or is restored, highlighting the role of early recognition and targeted interventions. Understanding the prevalence and diversity of motor manifestations in CKD is therefore essential for timely diagnosis, effective management, and prevention of long-term complications [1].

### 4.1. Specific Movement Disorders in CKD

#### 4.1.1. Restless Leg Syndrome

Restless legs syndrome (RLS), also termed Willis–Ekbom disease, represents one of the most prevalent movement disorders in patients with chronic kidney disease, with reported prevalence rates ranging from 20% to 40%, which is substantially higher than in the general population [1,4]. Clinically, RLS in CKD is characterized by an uncontrollable urge to move legs, typically accompanied by unpleasant sensory disturbances that are worse during periods of rest and at night, resulting in significant sleep disruption and impaired quality of life [27]. In haemodialysis patients, symptoms often intensify during or immediately after dialysis sessions, suggesting an interaction between rapid metabolic shifts and neuronal excitability [28]. It has been reported that RLS can reduce adherence to dialysis schedules due to nighttime discomfort in some cases [29]. Despite its high prevalence and impact on morbidity, RLS remains underrecognized in nephrology practice, highlighting the importance of routine screening and targeted management to improve sleep quality, daily functioning, and overall patient well-being.

#### 4.1.2. Myoclonus

Myoclonus is a clinically significant movement disorder observed in patients with CKD, particularly in advanced stages, with a prevalence reported at around 25% of patients [4]. It is characterized by sudden, brief, involuntary muscle jerks that may be focal, multifocal, or generalized, and can occur spontaneously or be triggered by voluntary movement or sensory stimuli [30]. While often mild, myoclonus can disrupt sleep, impair motor function, and negatively affect quality of life. Its prevalence and severity correlate with the degree of renal impairment, reflecting systemic metabolic derangements and the accumulation of uremic toxins [30].

#### 4.1.3. Flapping Tremor

Flapping tremor is a characteristic manifestation of metabolic encephalopathy in advanced CKD [31]. It is defined by intermittent, involuntary lapses in postural muscle tone, most readily observed as flapping of the hands during wrist extension [32]. The presence and severity of flapping tremor correlate with the degree of uraemia and systemic metabolic derangements [33]. While often subtle, pronounced flapping tremor may impair manual dexterity and signal impending neurological deterioration. Therapeutic focus is directed toward correction of underlying metabolic abnormalities and optimization of renal function [33].

#### 4.1.4. Periodic Limb Movements in Sleep

Periodic limb movements in sleep (PLMSs) are repetitive, stereotyped extensions and flexions of the lower limbs occurring during non-REM sleep [34]. PLMS is highly prevalent in patients with advanced CKD and frequently coexists with restless legs syndrome [35]. These movements disrupt sleep architecture, leading to fragmented sleep and excessive daytime somnolence. Their occurrence has been associated with impaired quality of life and may contribute to cardiovascular stress through repeated nocturnal arousals [35].

#### 4.1.5. Parkinsonism

Parkinsonism in CKD is uncommon but clinically significant, often resulting from uremic neurotoxicity, cerebrovascular lesions, or drug-related effects [36]. It presents with the classical triad of bradykinesia, rigidity, and resting tremor, often accompanied by postural instability [37]. Unlike idiopathic Parkinson’s disease, the severity and progression of symptoms may fluctuate with changes in metabolic and renal status. Recognizing Parkinsonian features in CKD is essential to distinguish reversible metabolic or drug-induced forms from primary neurodegenerative Parkinson’s disease [38].

#### 4.1.6. Peripheral Neuropathy

Peripheral neuropathy is the common complication of CKD, predominantly manifesting as a distal, symmetric sensorimotor polyneuropathy [39]. Importantly, it has been examined to occur in patients with CKD even in the absence of diabetes mellitus, indicating that this condition can develop independently of diabetes [40]. Clinical features include numbness, paraesthesia, and burning pain in the feet and hands, which are often exacerbated at night [40,41]. Electrophysiological studies demonstrate slowed nerve conduction and reduced amplitudes consistent with axonal involvement [39]. The severity of neuropathy correlates with the degree of renal impairment, reflecting systemic metabolic derangements inherent to CKD [39,42] (Table 1).

### 4.2. Impact of MDs on Quality of Life and Clinical Outcomes in CKD Patients

Movement disorders in CKD profoundly compromise physical function, independence, and overall well-being [43,44]. Impaired motor control, postural instability, and reduced coordination substantially increase the risk of falls, fractures, and related hospitalizations, particularly among older or frail patients [45,46]. Chronic neuromuscular deficits, including peripheral neuropathy and myoclonus, further restrict mobility and contribute to progressive deconditioning, sarcopenia, and diminished exercise tolerance [30,47]. These limitations often create a cycle of inactivity, further weakening musculoskeletal integrity and increasing vulnerability to secondary complications such as joint contractures and reduced bone density [48]. Sleep-disrupting conditions, notably restless legs syndrome and periodic limb movements, exacerbate daytime fatigue, impair cognitive function, and increase the incidence of mood disturbances, including depression and anxiety, which collectively reduce patients’ capacity for self-care, adherence to treatment regimens, and engagement in social or occupational activities [49,50]. Beyond functional limitations, movement disorders in CKD have substantial systemic consequences. Recurrent sleep fragmentation and associated sympathetic overactivity contribute to elevated cardiovascular risk, including nocturnal hypertension or arrhythmias, which may accelerate the progression of cardiovascular comorbidities already prevalent in CKD [51]. Persistent immobility, arising from neuropathy, myoclonus, or Parkinsonian features, promotes chronic inflammation, impaired microcirculation, thromboembolic risk, and susceptibility to pressure-related injuries [52]. Additionally, the combination of chronic fatigue, pain, and neuromotor impairment can exacerbate cognitive decline, reduce resilience to acute illness, and prolong recovery times, compounding overall morbidity [43]. Collectively, the physical, cognitive, and systemic burdens imposed by movement disorders in CKD substantially diminish functional independence, quality of life, and survival prospects, highlighting their importance in possible clinical trials [10,44] (Table 2).

## 5. Therapeutic Approaches

Management of MDs in CKD relies on both optimizing kidney function and targeted symptomatic therapies. Correction of uraemia and metabolic derangements (through intensified dialysis or kidney transplantation) often alleviates symptoms [1]. In parallel, non-pharmacological measures, including individualized exercise training and nutrition optimization, support motor function and improve quality of life [55,56]. For example, studies show that aerobic and resistance exercise during dialysis can significantly reduce the severity of RLS and improve overall physical capacity [55,56]. These general principles of care form the foundation for treating CKD-related neuromuscular MDs.

### 5.1. RLS and PLMS

RLS in CKD is managed similarly to idiopathic RLS, but with dose adjustments and attention to renal clearance. Dopaminergic agents (pramipexole, ropinirole, levodopa) and α2δ calcium-channel ligands (gabapentin, pregabalin) have been shown to reduce RLS symptoms in dialysis patients [3,57]. For instance, in an article focusing on CKD patients it was found that ropinirole significantly alleviated RLS severity and improved sleep without major side effects, while gabapentin and levodopa also provided benefit but with more adverse events [57]. Intravenous iron therapy is indicated in CKD-RLS with iron deficiency; placebo-controlled trials demonstrated that intravenous iron infusions significantly improve RLS scores (at least transiently) in haemodialysis patients [58,59]. Additional therapies include administration of vitamins C and E (which modestly reduced symptoms in small trials [60]) and non-drug interventions such as intradialytic exercise programs, physiotherapeutic massage, and improved sleep. All these approaches have been reported to decrease RLS/PLMS burden [3,56]. PLMS (often coexisting with RLS) are typically addressed by treating underlying RLS and, when needed, low-dose clonazepam or gabapentin at night to suppress leg jerks, although high-quality CKD-specific trials are lacking.

### 5.2. Myoclonus and Flapping Tremor

Uremic myoclonus reflects diffuse metabolic encephalopathy, and management concentrates on intensifying dialysis and correcting electrolyte or toxin abnormalities [3]. In cases of persistent or disruptive jerking, benzodiazepines (especially clonazepam) or valproate can be used to suppress myoclonic movements (drawing on general neurology practice). Thus, aggressive removal of uremic toxins (e.g., via more frequent dialysis or targeting urea, potassium and other solute levels) often leads to gradual resolution of myoclonus [13]. Flapping tremor also as a manifestation of advanced uremic encephalopathy, is not treated with specific drugs; rather, therapeutic methods focus on correction of underlying metabolic abnormalities and optimization of kidney function [33]. In practice, this means intensifying dialysis or expediting transplantation. As renal function improves, the characteristic flapping tremor typically subsides [33]. No trials of medications for flapping tremor in CKD have been reported to our knowledge so far, so management remains supportive and aimed at the underlying cause.

### 5.3. Parkinsonism

Parkinsonian symptoms in CKD must be distinguished into reversible (uremic or drug-induced) and primary forms. In reversible cases (e.g., diabetic uremic syndrome with bilateral basal ganglia lesions), treatment includes aggressive dialysis plus symptomatic dopaminergic therapy [61]. Case reports and series have documented that introducing levodopa or carbidopa concurrent with improved dialysis can cause an alleviation in rigidity and bradykinesia. Dopamine agonists may also be applied in the treatment, but evidence is sparse [61]. Vascular Parkinsonism in CKD is managed by addressing cerebrovascular risk factors and using standard therapies (physiotherapy, levodopa trials) cautiously [9]. In all cases, clinicians should avoid medications that could potentially worsen Parkinsonian features (e.g., metoclopramide) and monitor renal dosing closely [3].

### 5.4. Peripheral Neuropathy

Uremic neuropathy is usually treated by optimizing dialysis and correcting other contributing factors such as anaemia. Renal transplantation often stops progression or reverses neuropathy. Pharmacological therapy focuses on symptomatic relief of neuropathic pain, dose-adjusted gabapentin or pregabalin can ameliorate burning paraesthesia [55]. Other options include duloxetine or low-dose amitriptyline if tolerated. Supportive measures, which include tight glycaemic control in diabetics, potassium restriction to avoid further nerve injury, and supervised exercise/physical therapy, have shown benefit for neuropathic symptoms and overall mobility [55].

### 5.5. Address the Underlying Etiology of CKD

An essential strategy for slowing the progression of chronic kidney disease (CKD) is the effective management of its underlying etiology. In this context, biological agents have emerged as a promising and rapidly evolving class of therapies that provide targeted immunomodulation rather than generalized immunosuppression. Rituximab, a monoclonal anti-CD20 antibody, has demonstrated efficacy in the treatment of membranous nephropathy, ANCA-associated vasculitis, and refractory lupus nephritis by selectively depleting pathogenic B lymphocytes and reducing autoantibody production [56]. Complement inhibitors, including eculizumab and ravulizumab, target the terminal complement component C5 and have been shown to prevent complement-mediated endothelial and glomerular injury in atypical hemolytic uremic syndrome (aHUS), thereby preserving renal function and reducing systemic complications [57]. Belimumab, an anti-B-cell activating factor (BAFF) monoclonal antibody, is effective in lupus nephritis by modulating B-cell hyperactivity while sparing non-pathogenic immune cells, resulting in improved renal outcomes with a lower risk of systemic toxicity. Collectively, these biologic therapies exemplify the paradigm shift toward precision medicine in nephrology, offering mechanism-specific interventions that not only improve renal survival but also minimize adverse effects associated with traditional cytotoxic agents. Ongoing research continues to expand the therapeutic landscape, with the goal of optimizing treatment selection based on disease subtype, immunological profile, and individual patient characteristics.

### 5.6. Treatment Methods—Summary

Overall, in all MDs, a multimodal approach is required: restoring kidney function and removing toxins underlies all therapies [3], while pharmacological agents (dopaminergic, gabapentinoids, benzodiazepines, etc.) and rehabilitation strategies (exercise, sleep interventions) are applied according to the specific motor disorder. This integrated strategy has improved symptoms in studies of CKD patients (for example, dialysis-based exercise reduced RLS severity [56] and dopaminergic drugs eased uremic RLS [3]), but many questions remain about optimal dosing and long-term outcomes. Consistent with clinical guidelines, individualization of therapy, balancing benefits with side effects in the context of renal impairment, is essential for maximizing patient function and quality of life.

## 6. Current Knowledge Gaps and Directions for Future Research

Despite growing recognition of motor disturbances in CKD, the underlying mechanisms, although partially identified, remain insufficiently characterized. For instance, while recent studies suggest that CKD increases blood–brain barrier (BBB) permeability and promotes neuroinflammation, the exact mediators (e.g., indoxyl sulphate, guanidino compounds, advanced glycation end-products) and their neuronal targets (basal ganglia, motor cortex, peripheral nerves, neuromuscular junction) have yet to be comprehensively examined and identified [16,62].

Emerging evidence indicates that aldosterone and other hormonal imbalances in CKD may compromise BBB integrity, thereby impairing neurovascular function [63]. Moreover, in CKD transporters such as OAT3, which normally help remove uremic toxins from the brain, may be disrupted. However, the precise mechanisms of that phenomenon remain poorly understood [64]. Dedicated mechanistic studies, using both animal models and human tissues, are needed to determine how specific uremic solutes, oxidative stress, and endothelial dysfunction contribute to motor neuron injury or synaptic loss. Such research could identify novel therapeutic targets and help explain why certain CKD patients develop various MDs, while others remain unaffected [63,65].

Advanced neuroimaging and biomarker studies offer a promising way to address these knowledge gaps. Large longitudinal brain imaging studies are needed in CKD to directly link the progression of renal decline with structural and functional changes in the brain [66]. Longitudinal MRI studies could track the progression of white-matter microvascular disease or cortical atrophy in CKD cohorts, while PET imaging with amyloid, tau, or dopaminergic tracers could help detect concurrent neurodegenerative pathologies [66]. In parallel, fluid biomarkers such as neurofilament light chain (NfL), GFAP, and phosphorylated tau are elevated in CKD and show a strong inverse correlation with GFR [67]. However, their interpretation may be complicated by reduced renal clearance. Rigorous studies are needed to clarify whether increases in NfL or other markers truly predict motor or cognitive decline in CKD [67]. Combining imaging and biochemical markers (for example, pairing diffusion-tensor MRI with plasma NfL or oxidative-stress metabolites) could elucidate the pathways linking kidney failure to brain and neuromuscular injury [66,68].

Future clinical trials should focus on early detection and tailored interventions. Development and validation of screening tools, such as instrumented gait analysis, balance testing, or sensor-based motor assessments, could help identify CKD patients at risk for neuromuscular decline before overt disability occurs [69]. For example, pilot data show that not necessarily only 5 stage CKD patients exhibit altered gait variability and dual-task costs, suggesting the need for simple clinic-based tests [69]. Exercise and rehabilitation trials are also needed. Although consensus statements acknowledge that regular aerobic and resistance exercise can improve strength and endurance in CKD [70] implementation is inconsistent. Randomized trials should test structured exercise regimens (e.g., combined aerobic plus strength training, balance/gait retraining) specifically in CKD populations, with protocols individualized by CKD stage and comorbidity. Non-pharmacological interventions like cognitive–motor dual-task training or virtual reality balance programs are other promising areas. Additionally, neuroprotective strategies deserve investigation: for instance, agents that improve the function of the BBB (e.g., mineralocorticoid antagonists like spironolactone, which preserves BBB integrity in CKD models [63]) or therapies that reduce uremic toxin burden (such as adsorbents or optimized dialysis regimens) could be trailed for neurological benefit.

Addressing these gaps will require coordinated, multidisciplinary effort. Nephrologists, neurologists, neuroimaging experts, and rehabilitation specialists should collaborate on study design and care models. For instance, comprehensive renal rehabilitation clinics that integrate kidney care with physical and occupational therapy could both deliver interventions and serve as platforms for research. Engaging such teams, along with primary care and endocrinology (given diabetes’ role), would be crucial to translate findings into practice. In summary, a concerted research agenda focusing on advanced imaging, mechanistic studies, biomarker validation, and targeted interventions is needed to improve understanding and management of CKD-related motor disorders.

## 7. Conclusions

In conclusion, motor disorders are common but underrecognized complications of chronic kidney disease, arising from multifactorial mechanisms including uremic toxicity, vascular injury, and neuroinflammation. They manifest most frequently as restless legs syndrome, peripheral neuropathy, myoclonus, flapping tremor, periodic limb movements, and, less often, parkinsonism, with prevalence rates markedly higher than in the general population (RQ1). These disturbances contribute to impaired mobility, insomnia, diminished quality of life, and are strongly linked to increased risks of falls, cardiovascular events, and mortality (RQ2). Effective management requires not only correction of metabolic derangements but also targeted therapies; pharmacological agents may alleviate symptoms but are limited by renal dosing and adverse effects, while structured exercise and rehabilitation programs consistently improve functional capacity and quality of life, highlighting the value of multimodal strategies (RQ3). Early recognition and integrated, multidisciplinary interventions appear essential for slowing or preventing progression of motor dysfunction, though current evidence on long-term outcomes remains limited and underscores the need for robust CKD-specific trials (RQ4). Future research should prioritize systematic screening, mechanistic investigations, and individualized interventional studies to reduce the burden of these disorders and improve patient outcomes.

## Figures and Tables

**Table 1:** Summary of studies exploring the role of movement disorders in chronic kidney disease.

Author, Year, Reference	Movement Disorder(s) Studied	Key Findings
Mahmood et al. (2023), [4]	RLS, myoclonus, flapping tremor, PLMS, Parkinsonism	The prevalence of MDs in patients with CKD is high (28.7%, 24.1%, 20.9%, 17% and 2.8% for RLS, myoclonus, flapping tremor, PLMS and Parkinsonism, respectively.) Longer dialysis periods increase the risk for PLMS and Parkinsonism.
Jerzak et al. (2025), [7]	Coordination disorders assessed by Short Physical Performance Assessment [S-PPA]	Higher S-PPA Scores in patients with CKD are linked to more falls, cardiovascular events, and deaths
Safarpour et al. (2023), [3]	RLS	RLS occurs two to three times more often in individuals with CKD than in the general population. In patients with CKD, RLS is associated with higher mortality, cardiovascular complications, depression, sleep disturbances, and reduced quality of life.
Safarpour et al. (2021), [1]	RLS, myoclonus, flapping tremor, dystonia, chorea, tremor, Parkinsonism	The most common movement disorders observed in CKD are RLS, myoclonus, and flapping tremor. The severity of these conditions usually alleviates in parallel with the restoration of renal function.
Krishnan et al. (2009), [5]	Neuromuscular disease (reduced exercise capacity, weakness, disability)	Almost all dialysis patients suffer from neuromuscular disease. Potassium retention may contribute to the development of neuropathy. Increased dialysis frequency can improve the condition.
Roshanravan et al. (2017), [10]	Muscle dysfunction, frailty, mobility disability	CKD contributes to muscle dysfunction and frailty. Early detection combined with personalized exercise programs can help prevent declines in quality of life and the development of disability.
Arnold et al. (2019), [6]	Peripheral neuropathy	Peripheral neuropathy is highly prevalent in CKD, including non-diabetic patients. Proper potassium management may improve neuromuscular outcomes.

**Table 2:** Movement disorders and their impact on quality of life in patients with CKD.

Movement Disorder	Prevalence in CKD	Key Clinical Features	Impact on Quality of Life	References
RLS	The prevalence varies across the studies in range 20–40% of CKD patients. The prevalence is usually reported as 2–3 times higher than in general population.	Compelling urge to move the legs, predominantly during rest and nocturnal hours	Chronic insomnia, daytime somnolence and cognitive impairment, increased risk of mood disorders (e.g., depression)	[4,53]
PLMS	Rates are reported from 17% to even 60% in CKD patients.	Repetitive, stereotyped dorsiflexion movements of the ankles or legs during sleep. Usually occur in clusters, often associated with micro-arousals	Sleep fragmentation and reduced sleep efficiency, fatigue, impaired cognitive performance	[4,50]
Parkinsonism	The prevalence rates are rather low and range at around 3% of patients with CKD.	Bradykinesia, muscular rigidity, and resting tremor. Postural instability with impaired balance and coordination. Gait disturbances, including shuffling steps and freezing episodes. Cognitive decline and neuropsychiatric symptoms (depression, anxiety, apathy)	Increased risk of falls, fractures, and related morbidity, Progressive loss of independence in activities of daily living, Reduced mobility leading to social isolation and decreased physical activity	[4]
flapping tremor	Rates are reported at around 21% of CKD patients.	Sudden, brief lapses in sustained posture with flapping movements, most evident in the hands and wrists, typically elicited during wrist extension or dorsiflexion of the hands	Functional disability due to impaired fine motor control and difficulty performing daily activities. Contributes to dependence on caregivers and loss of autonomy	[4]
Neuromuscular disease	The condition is prevalent in end stages of renal disease and affects almost all dialysis patients.	Weakness, reduced exercise capacity, disability	Difficulty walking, balance issues, increased fall risk. Limits independence in daily activities; difficulty with self-care	[5]
Myoclonus	Myoclonus can affect around ¼ of people suffering from CKD, especially in advanced stages	Sudden, involuntary muscle jerks, Muscle stiffness or spasms, Impaired coordination	Difficulty with walking, balance, and precise movements. Reduces ability to perform physical tasks, affects mood	[4]
Peripheral neuropathy	Prevalence rates range from approximately 50–60% of patients with end-stage renal disease to even 70% in dialysis patients.	Gait abnormalities (foot drop, unsteady gait), Impaired reflexes, Progressive functional decline	Loss of mobility, increased fall risk, social withdrawal, Slowed motor response, difficulty with balance and coordination. Higher amputation risks	[53,54]

## Data Availability

No new data were created or analyzed in this study.

## Abbreviations

The following abbreviations are used in this manuscript:

RLS	Restless legs syndrome
CKDs	Chronic Kidney Diseases
MDs	Motor disorders
IS	indoxyl sulphate
NfL	neurofilament light chain
BBB	blood–brain barrier
PLMS	Periodic limb movements in sleep
MSNA	muscle sympathetic nerve activity
PCS	p-cresol sulphate
ROS	reactive oxygen species
aHUS	atypical hemolytic uremic syndrome
